# An enhancement of binary particle swarm optimization for gene selection in classifying cancer classes

**DOI:** 10.1186/1748-7188-8-15

**Published:** 2013-04-24

**Authors:** Mohd Saberi Mohamad, Sigeru Omatu, Safaai Deris, Michifumi Yoshioka, Afnizanfaizal Abdullah, Zuwairie Ibrahim

**Affiliations:** 1 Artificial Intelligence and Bioinformatics Research Group, Faculty of Computer Science and Information Systems, Universiti Teknologi Malaysia, Skudai, Johor, 81310, Malaysia; 2 Department of Electronics, Information and Communication Engineering, Osaka Institute of Technology, Osaka, 535-8585, Japan; 3 Department of Computer Science and Intelligent Systems, Graduate School of Engineering, Osaka Prefecture University, Sakai, Osaka, 599-8531, Japan; 4 Faculty of Electrical and Electronic Engineering, Universiti Malaysia Pahang, Pekan, Pahang, 26600, Malaysia

## Abstract

**Background:**

Gene expression data could likely be a momentous help in the progress of proficient cancer diagnoses and classification platforms. Lately, many researchers analyze gene expression data using diverse computational intelligence methods, for selecting a small subset of informative genes from the data for cancer classification. Many computational methods face difficulties in selecting small subsets due to the small number of samples compared to the huge number of genes (high-dimension), irrelevant genes, and noisy genes.

**Methods:**

We propose an enhanced binary particle swarm optimization to perform the selection of small subsets of informative genes which is significant for cancer classification. Particle speed, rule, and modified sigmoid function are introduced in this proposed method to increase the probability of the bits in a particle’s position to be zero. The method was empirically applied to a suite of ten well-known benchmark gene expression data sets.

**Results:**

The performance of the proposed method proved to be superior to other previous related works, including the conventional version of binary particle swarm optimization (BPSO) in terms of classification accuracy and the number of selected genes. The proposed method also requires lower computational time compared to BPSO.

## Background

Scientists are allowed to measure the expression levels of thousands of genes simultaneously in the field of biological organisms by utilizing recent advancement in microarrays technology. This technology allows and produces databases of cancerous tissues based on gene expression data [1]. The output of the microarrays technology are gene expression data which consist of valuable information of genomic, diagnostic, and prognostic for researchers. Thus, it is necessary to select informative genes that are really related to cancerous state [2]. However, the gene selection process normally faces problems due to the subsequent features of gene expression data: the large number of genes compared to the small number of samples (high-dimensional data), irrelevant genes, and noisy data. Gene selection is called feature selection in artificial intelligence domain. The gene selection has several advantages [3]:

1) keeps or enhances classification accuracy.

2) minimizes dimensionality of the data.

3) reduces computational time.

4) eliminates irrelevant and noisy genes.

5) minimizes the cost in a clinical setting.

Gene expression analysis has been intensively researched for more than a decade [4]. Selecting a smaller subset of informative genes from thousands of genes is a crucial step for accurate cancer classification based on gene expression data. Gene selection methods can be classified into two categories in the context of machine learning [3]. A gene selection method is categorized as a filter method if it is carried out independently from a classification procedure. Otherwise, it is called as a hybrid (wrapper) method. Most previous works in the early era of microarrays analysis have used the filter method to select genes due to its lower computation compared to the hybrid method. T-test, signal-to-noise-ratio, information gain, etc. are the examples of the filter method that are usually considered as individual gene-ranking methods. Researchers evaluate a gene based on its discriminative power for the target classes without considering its correlations with other genes, which may result in the inclusion of irrelevant and noisy genes in a gene subset for cancer classification. These genes increase the dimensionality of the gene subset and, in turn affect the classification performance. The filter methods also select a number of genes manually, which is known having difficulties in usage, especially for beginner biologists. It has been demonstrated that genes in a cell do not act independently. They interact with one another to complete certain biological processes or to implement certain molecular functions [5].

Therefore, several hybrid methods, especially combinations between particle swarm optimization (PSO) and a classifier, have been implemented to select informative genes in since few years ago up to recent time [3,6-9]. The hybrid methods usually produce better result in term of accuracy than the filter methods, since the genes are selected by considering and optimizing the correlations among genes. PSO is a new population based stochastic optimization technique. It was proposed by Kennedy and Eberhart [10] based on the observation of social behavior of organisms such as bird flocking and fish schooling. PSO has been successfully implemented in many researches and application areas. It also has only few parameters to adjust and is easier to apply in many applications.

Shen *et al*. [3] introduced a hybrid of PSO and tabu search approaches for gene selection. Unfortunately, the results obtained by using the hybrid method were less meaningful because the tabu approaches in PSO were unable to search for a near-optimal solution in search spaces. Next, Chuang *et al.* proposed an improved binary PSO [6]. Using the proposed method yields 100% classification accuracy in many data sets, but it utilizes a large number of selected genes (large gene subset) to obtain the high accuracy. This method uses a large number of genes because the global best particle is reset to the zero position when its fitness values do not change after three consecutive iterations. Li *et al*. brought out an idea of hybrid of PSO and genetic algorithms (GA) for the same purpose thereafter [7]. Unfortunately, the result still did not pose high accuracy and many genes had to be selected for cancer classification because there were no direct probability relations between GA and PSO. Chuang *et al*. [8,9] also introduced a combination of tabu search and PSO for gene selection, and currently they proposed a hybrid of BPSO and a combat GA for the same purpose. However, the both proposed approaches [8,9] still need a high number of selected genes to result high classification accuracy. A significant weakness is also found because of a combination of PSO and a tabu search technique, and a hybrid of BPSO and a combat GA which do not share probability significance in their processes. Generally, the PSO-based methods [3,6-9] are incapable to yield a small (near-optimal) subset of informative genes for high classification accuracy. This drawback is known caused mainly by the large number of gene (high-dimension data).

Accurate classification of microarray data is critical for successful clinical diagnosis and treatment [11]. Thus, one diagnostic goal aims to develop a medical procedure based on the least number of genes that are needed to detect diseases accurately. We propose an enhanced binary PSO (EPSO) to select a small (near-optimal) subset of informative genes that is most relevant for cancer classification. To test the effectiveness of our proposed method, we applied the EPSO to ten gene expression data sets, including binary-classes and multi-classes data sets.

This paper is organized as follow. In Section 2, we briefly describe the conventional version of binary PSO and EPSO. Section 3 presents the data sets used and the experimental results. Section 4 summarizes this paper by providing its main conclusions and addresses future directions.

## Methods

### A conventional version of binary PSO (BPSO)

Binary PSO (BPSO) is initialized with a population of particles. All particles move in a problem space to find the optimal solution for each of the iteration. A potential solution in an *n*-dimensional space is presented by a particle. Each particle has its own position and velocity vectors for guiding its movement. The position and velocity vectors of the *i*th particle in the *n*-dimension can be represented as *X*_*i*_ = (*x*_*i*_^1^, *x*_*i*_^2^,…, *x*_*i*_^*n*^) and *V*_*i*_ = (*v*_*i*_^1^, *v*_*i*_^2^, …, *v*_*i*_^*n*^), respectively, where *x*_*i*_^*d*^ ∈ {0, 1}; *i*=1,2,..*m* (*m* is the total number of particles) and *d*=1,2,..*n* (*n* is the dimension of data) [12].

*v*_*i*_^*d*^ is a real number for the *d-*th dimension of the particle *i*, where the maximum *v*_*i*_^*d*^ is *V*_max_ = (1/3) × *n*. This maximum value is important, as it controls the granularity of the search by clamping the escalating velocities. A large value of *V*_max_ assists the global exploration, while a small value encourages the local exploitation. If *V*_max_ is too small, the swarm may not be able to explore sufficiently beyond locally good regions. In addition, the small value of *V*_max_ increases the number of time steps to reach an optimum and may become trapped in a local optimum. On the other hand, the possibility of missing a good region becomes higher if values of *V*_max_ is too large. The particles continue to search in the gainless regions of the search space since they may escape from the good solutions. In our preliminary experiment, we have found that an appropriate maximum velocity value (1/3) × *n* was found after a several preliminary runs. We chose *V*_max_ = (1/3) × *n* and limited the velocity within the range [1, (1/3) × *n*] which prevented an overly large velocity. A particle can be close to an optimal solution, but a high velocity may make it move far away. By limiting the maximum velocity, particles cannot fly too far away from the optimal solution. Therefore, the BPSO method has a greater chance to find the optimal solution under the limit.

For gene selection, a binary bit string of length *n* represents the vector of particle where *n* is the total number of genes. Each position vector (*X*_*i*_) represents a gene subset. The corresponding gene is selected if the value of the bit is 1. On the other hand, the gene is not selected if the value is 0. The following equations show how each particle in the *t*-th iteration updates its own position and velocity:

(1) $$\begin{array}{l} v_{i}^{d}\left(t+1\right)=w\left(t\right)\times v_{i}^{d}\left(t\right)+c_{1}r_{1}^{d}\left(t\right)\\ \;\times \left(\text{pbes}t_{i}^{d}\left(t\right)-x_{i}^{d}\left(t\right)\right)+c_{2}r_{2}^{d}\left(t\right)\\ \;\times \left(\text{gbes}t^{d}\left(t\right)-x_{i}^{d}\left(t\right)\right) \end{array}$$

(2) $$\text{Sig}\left(v_{i}^{d}\left(t+1\right)\right)=\frac{1}{1+e^{-v_{i}^{d}\left(t+1\right)}}$$

if *Sig*(*v*_*i*_^*d*^(*t* + 1)) > *r*_3_^*d*^(*t*), then *x*_*i*_^*d*^(*t* + 1) = 1; or otherwise

(3) $$x_{i}^{d}\left(t+1\right)=0$$

where *c*_1_ and *c*_2_ are the acceleration constants in the interval [0,2] and $r_{1}^{d}\left(t\right),r_{2}^{d}\left(t\right),r_{3}^{d}\left(t\right)\sim U\left(0,1\right)$ are random values in the range [0,1], which are sampled from a uniform distribution. *Pbest*_*i*_(*t*) = (*pbest*_*i*_^1^(*t*), *pbest*_*i*_^2^(*t*), …, *pbest*_*i*_^*n*^(*t*)) and *Gbest*(*t*) = (*gbest*^1^(*t*), *gbest*^2^(*t*), …, *gbest*^*n*^(*t*)) signify the best previous position of the *i*th particle and the global best position of the swarm (all particles), respectively. They are evaluated by a fitness function. *Sig*(*v*_*i*_^*d*^(*t* + 1)) is a sigmoid function where *Sig*(*v*_*i*_^*d*^(*t* + 1)) ∈ [0, 1]. *w*(*t*) is an inertia weight, which was introduced by Shi and Eberhart [13] as a mechanism to control the exploration and exploitation abilities of the swarm and remove the need for velocity clamping. The momentum of the particle is controlled by the inertia weight through weighting the contribution of the previous velocity, namely controlling how much memory of the previous particle direction will influence the new velocity. A nonlinear decreasing approach was applied in BPSO to update *w*(*t*) in each of the iteration. An initially large value decreased nonlinearly to a small value in this approach. It also permitted a shorter exploration time than a linear decreasing approach where more time would be spent on refining solution (exploiting). The *w*(*t*) was initialized with a value of 1.4 and was updated as follows [14,15]:

(4) $$w\left(t+1\right)=\frac{\left(w\left(t\right)-0.4)\times (\text{MAXITER}-\text{Iter}\left(t\right)\right)}{\left(\text{MAXITER}+0.4\right)}$$

where *MAXITER* is the maximum iteration (generation) and *Iter*(*t*) is the current iteration. The pseudo code of BPSO described in the following section.

### The Pseudo code of BPSO

The pseudo code of BPSO as follows:

### Investigating the drawbacks of BPSO and previous PSO-based methods

It would be practical to find the limitations of BPSO and previous PSO-based methods before attempting to propose EPSO [3,6-9]. This subsection investigates the limitations of these methods by analyzing Eq. 2 and Eq. 3, which are the most crucial equations for gene selection in binary spaces. Both these equations are also implemented in BPSO and the PSO-based methods.

The sigmoid function (Eq. 2) represents a probability for *x*_*i*_^*d*^(*t*) to be 0 or 1 (*P*(*x*_*i*_^*d*^(*t*) = 0) or *P*(*x*_*i*_^*d*^(*t*) = 1)). For example,

if *v*_*i*_^*d*^(*t*) = 0, then *Sig*(*v*_*i*_^*d*^(*t*) = 0) = 0.5 and *P*(*x*_*i*_^*d*^(*t*) = 0) = 0.5.

if *v*_*i*_^*d*^(*t*) < 0, then *Sig*(*v*_*i*_^*d*^(*t*) < 0) < 0.5 and *P*(*x*_*i*_^*d*^(*t*) = 0) > 0.5.

if *v*_*i*_^*d*^(*t*) > 0, then *Sig*(*v*_*i*_^*d*^(*t*) > 0) > 0.5 and *P*(*x*_*i*_^*d*^(*t*) = 0) < 0.5.

In addition, *P*(*x*_*i*_^*d*^(*t*) = 0) = 1 − *P*(*x*_*i*_^*d*^(*t*) = 1). Thus, we concluded that *P*(*x*_*i*_^*d*^(*t*) = 0) = *P*(*x*_*i*_^*d*^(*t*) = 1) = 0.5 for the initial iteration, because Eq. 2 is a standard sigmoid function without any constraint and no modification. Although the next iterations potentially influence the *P*(*x*_*i*_^*d*^(*t*) = 0) or *P*(*x*_*i*_^*d*^(*t*) = 1), the *P*(*x*_*i*_^*d*^(*t*) = 0) = *P*(*x*_*i*_^*d*^(*t*) = 1) = 0.5 mostly maintained its application on the gene expression data because the gene expression data was highly dimensional and had a large search space. It only minimized the number of genes to about half of the total number of genes by using the standard sigmoid function in high-dimensional data. This is shown and proven in the section of experimental results. Therefore, Eq. 2 and Eq. 3 could potentially be the shortcomings of BPSO and the previous PSO-based methods in selecting a small number of genes for producing a near-optimal (small) subset of genes from the gene expression data.

### An enhanced binary PSO (EPSO)

In almost all previous gene expression data researches, a subset of genes is commonly selected for excellent cancer classifications. Thus, we propose EPSO for the selection of a near-optimal (small) subset of genes in order to overcome the shortcomings of BPSO and previous PSO-based methods [3,6-9]. EPSO differs from the BPSO and PSO-based methods in three ways: 1) we introduced a scalar quantity called particle speed (*s*); 2) we modified the existing sigmoid function; 3) we proposed a rule for updating *x*_*i*_^*d*^(*t* + 1). In contrast, the BPSO and PSO-based methods do not apply particle speed, use the standard sigmoid function (Eq. 2), and implement the original rule (Eq. 3). The particles’ speed, modified sigmoid function, and new rule were introduced in order to:

maximize the probability of *x*_*i*_^*d*^(*t* + 1) = 0 (*P*(*x*_*i*_^*d*^(*t* + 1) = 0)) and meanwhile

minimize the probability of *x*_*i*_^*d*^(*t* + 1) = 1 (*P*(*x*_*i*_^*d*^(*t* + 1) = 1)).

A small number of genes were selected and grouped into a gene subset caused by the increased and decreased probability values. *x*_*i*_^*d*^(*t* + 1) = 1 represents that the corresponding gene is selected. Otherwise, *x*_*i*_^*d*^(*t* + 1) = 0 indicates that the corresponding gene is not selected.

**Definition 1.***s*_*i*_*is the speed, length or magnitude of V*_*i*_*for the particle i. Therefore, the following properties of s*_*i*_*are crucial:*

*non-negativity: s*_*i*_ ≥ 0;

*definiteness: s*_*i*_ = 0 *if and only if V*_*i*_ = 0;

*homogeneity:* ‖*αV*_*i*_‖ = *α*‖*V*_*i*_‖ = *αs*_*i*_*where α* ≥ 0;

*the triangle inequality:* ‖*V*_*i*_ + *V*_*i*+1_‖ ≤ ‖*V*_*i*_‖ + ‖*V*_*i*+1_‖ *where* ‖*V*_*i*_‖ = *s*_*i*_*and* ‖*V*_*i*+1_‖ = *s*_*i*+1_*.*

The particles’ speed (Equation 5), sigmoid function (Equation 6), and the rule (Equation 7) are proposed as follow:

(5) $$\begin{array}{c} s_{i}\left(t+1\right)=w\left(t\right)\times s_{i}\left(t\right)+c_{1}r_{1}\left(t\right)\\ \;\times \text{dist}\left(\text{pbes}t_{i}\left(t\right)-X_{i}\left(t\right)\right)+c_{2}r_{2}\left(t\right)\\ \;\times \text{dist}\left(\text{Gbest}\left(t\right)-X_{i}\left(t\right)\right) \end{array}$$

(6) $$\text{Sig}\left(s_{i}\left(t+1\right)\right)=\frac{1}{1+e^{-s_{i}\left(t+1\right)}}$$

subjected to *s*_*i*_(*t* + 1) ≥ 0 if *Sig*(*s*_*i*_(*t* + 1)) > *r*_3_^*d*^(*t*), then *x*_*i*_^*d*^(*t* + 1) = 0; or else

(7) $$x_{i}^{d}\left(t+1\right)=1$$

where *s*_*i*_(*t* + 1) represents the speed of the particle *i* for the *t*+1 iteration. By contrast, in BPSO and other PSO-based methods (Eq. 1, Eq. 2, and Eq. 3), *v*_*i*_^*d*^(*t* + 1) is related to a single element of a particle velocity vector for the particle *i*. In EPSO, Eq. 5, Eq. 6, and Eq. 7 were used to replace Eq. 1, Eq. 2, and Eq. 3, respectively. *s*_*i*_(*t* + 1) is the rate at which the particle *i* changes its position. *s*_*i*_(*t* + 1) ≥ 0 is the most significant property of *s*_*i*_(*t* + 1) based on Definition 1. Hence, *s*_*i*_(*t* + 1) was used instead of *v*_*i*_^*d*^(*t* + 1) so that its positive value could increase *P*(*x*_*i*_^*d*^(*t* + 1) = 0).

*s*_*i*_(*t* + 1) for each of the particle was initialized with positive real numbers in Eq. 5. The distance between *Pbest*_*i*_(*t*) and *X*_*i*_(*t*) (*dist*(*Pbest*_*i*_(*t*) − *X*_*i*_(*t*))), and the distance between *Gbest*(*t*) and *X*_*i*_(*t*) (*dist*(*Gbest*(*t*) − *X*_*i*_(*t*))), are the basic features that needed to be taken into consideration for calculating and updating *s*_*i*_(*t* + 1) in Eq. 5, whereas *v*_*i*_^*d*^(*t* + 1) was calculated by using the original formula (Eq. 1) and it was fundamentally based on the difference between *Pbest*_*i*_^*d*^(*t*) and *x*_*i*_^*d*^(*t*), and the difference between *Gbest*^*d*^(*t*) and *x*_*i*_^*d*^(*t*). The distances were used in the calculation for updating *s*_*i*_(*t* + 1) in order to make sure that Eq. 6 always satisfied the property of *s*_*i*_(*t* + 1), namely (*s*_*i*_(*t* + 1) ≥ 0) and to increase *P*(*x*_*i*_^*d*^(*t* + 1) = 0). The next subsection shows how to calculate the distance between two positions of two particles, e.g., *dist*(*Gbest*(*t*) − *X*_*i*_(*t*)).

Equations (5–7) and *s*_*i*_(*t*) ≥ 0 increase *P*(*x*_*i*_^*d*^(*t*) = 0) due to the minimum value for *P*(*x*_*i*_^*d*^(*t*) = 0) which was 0.5 when *s*_*i*_(*t*) = 0 ( min *P*(*x*_*i*_^*d*^(*t*) = 0) ≥ 0.5). In addition, they reduced the maximum value for *P*(*x*_*i*_^*d*^(*t*) = 1) to 0.5 ( max *P*(*x*_*i*_^*d*^(*t*) = 1) ≤ 0.5). Therefore, if *s*_*i*_(*t*) > 0, then *P*(*x*_*i*_^*d*^(*t*) = 0) > > 0.5 and *P*(*x*_*i*_^*d*^(*t*) = 1) < < 0.5.

**Figure 1:**
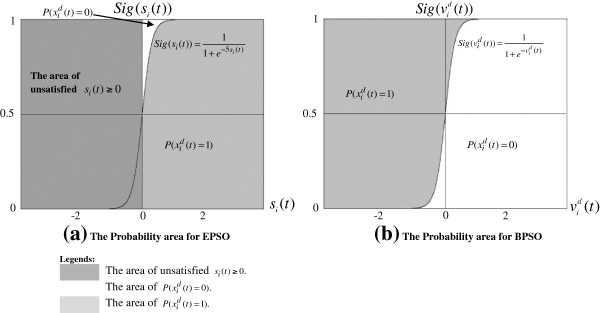
**The areas of unsatisfied *s***_***i***_**(*t*) ≥ 0,** ***P*(*x***_***i***_^***d***^**(*t*) = 0) and *P*(*x***_***i***_^***d***^**(*t*) = 1) in EPSO and BPSO.**

Figure 1 shows that a) Equations (5–7) and *s*_*i*_(*t*) ≥ 0 in EPSO increase *P*(*x*_*i*_^*d*^(*t*) = 0); b) Equations (1–3) in BPSO yield *P*(*x*_*i*_^*d*^(*t*) = 0) = *P*(*x*_*i*_^*d*^(*t*) = 1) = 0.5. As an example, the calculations for *P*(*x*_*i*_^*d*^(*t*) = 0) and *P*(*x*_*i*_^*d*^(*t*) = 1) in Figure 1(a) are shown as follow:

if *s*_*i*_(*t*) = 1, then *P*(*x*_*i*_^*d*^(*t*) = 0) = 0.993307 and *P*(*x*_*i*_^*d*^(*t*) = 1) = 1 − *P*(*x*_*i*_^*d*^(*t*) = 0) = 0.006693.

if *s*_*i*_(*t*) = 2, then *P*(*x*_*i*_^*d*^(*t*) = 0) = 0.999955 and *P*(*x*_*i*_^*d*^(*t*) = 1) = 1 − *P*(*x*_*i*_^*d*^(*t*) = 0) = 0.000045.

From the calculation above (based on Eq. 6), the modified sigmoid function (Eq. 6) generates higher *P*(*x*_*i*_^*d*^(*t*) = 0) compared to the standard sigmoid function (Eq. 2). For example, the calculations for *P*(*x*_*i*_^*d*^(*t*) = 0) and *P*(*x*_*i*_^*d*^(*t*) = 1) based on Eq. 2 in Figure 1(b) are shown as follow:

if *v*_*i*_^*d*^(*t*) = 1, then *P*(*x*_*i*_^*d*^(*t*) = 0) = 0.731059 and *P*(*x*_*i*_^*d*^(*t*) = 1) = 1 − *P*(*x*_*i*_^*d*^(*t*) = 0) = 0.268941.

if *v*_*i*_^*d*^(*t*) = 2, then *P*(*x*_*i*_^*d*^(*t*) = 0) = 0.880797 and *P*(*x*_*i*_^*d*^(*t*) = 1) = 1 − *P*(*x*_*i*_^*d*^(*t*) = 0) = 0.119203.

A small number of genes were selected in order to produce a near-optimal (small) gene subset from high-dimensional data (gene expression data) based on the high probability of *x*_*i*_^*d*^(*t*) = 0 (*P*(*x*_*i*_^*d*^(*t*) = 0)). Hence, EPSO is proposed to surmount the limitations of BPSO and the previous PSO-based methods and to produce a small subset of informative genes as a result.

### Calculating the distance of two particles’ positions

The number of different bits between two particles is related to the difference between their positions. For example, let us consider *Gbest*(*t*) = [0, 0, 1, 1, 1, 0, 1, 0, 0, 0] and *X*_*i*_(*t*) = [1, 1, 1, 0, 1, 1, 0, 1, 0, 0]. The difference between *Gbest*(*t*) and *X*_*i*_(*t*) is indicated as *diff*(*Gbest*(*t*) − *X*_*i*_(*t*)) = [ − 1, − 1, 0, 1, 0, − 1, 1, − 1, 0, 0]. A value of 1 indicates that this bit (gene) should be selected in comparison with the best position. However, if it is not selected, the classification quality may decrease and direct to a lower fitness value. In contrast, a value of −1 shows that this bit should not be selected in comparison with the best position, but it is nonetheless selected. The selection of irrelevant genes makes the length of the subset longer and leads to a lower fitness value. The number of 1 is assumed to be *a*, whereas the number of −1 is *b*. We used the absolute value of *a* − *b* (|*a* − *b*|) to express the distance between the two positions. In this example, the distance between *Gbest*(*t*) and *X*_*i*_(*t*) is *dist*(*Gbest*(*t*) − *X*_*i*_(*t*)) = |*a* − *b*| = |2 − 4| = 2.

### Fitness functions

The fitness value of a particle (a gene subset) is calculated as follows:

(8) $$\text{fitness}\left(X_{i}\right)=w_{1}\times A\left(X_{i}\right)+\left(w_{2}\left(n-R\left(X_{i}\right)\right)/n\right)$$

in which *A*(*X*_*i*_) ∈ [0, 1] is the leave-one-out-cross-validation (LOOCV) classification accuracy that uses the only gene in a gene subset (*X*_*i*_). Support vector machine classifiers (SVM) provide this accuracy. *R*(*X*_*i*_) is the number of selected genes in *X*_*i*_. *n* is the total number of genes for each sample. The importance of accuracy and the number of selected genes correspond to two priority weights *w*_1_ and *w*_2_, respectively. For this article, the accuracy is more important than the number of selected genes. Thus, we selected the value of *w*_1_ in the range [0.6, 0.9] and we set *w*_2_ = 1 − *w*_1_. In order to produce the remaining percentage of weights after the value of *w*_1_ had been chosen, the value of *w*_2_ was set to 1 − *w*_1_.

## Results and discussion

### Data sets and experimental setup

Table 1 summarizes the gene expression data sets used in this study. The data sets incorporated binary-classes and multi-classes data sets that have thousands of genes (high-dimensional data). They were downloaded from http://www.gems-system.org. All of the experimental results reported in this article were acquired using Rocks Linux version 4.2.1 (Cydonia) on the IBM xSeries 335 (a cluster machine that contains 13 compute nodes). Each compute node has four high performances 3.0 GHz Intel Xeon CPUs with 512 MB of memory. Thus, a total of 52 CPUs for the 13 compute nodes were used. The IBM xSeries 335 with 52 CPUs was needed to experiment EPSO and BPSO because both these methods have huge computational times and run on high-dimensional data. The computational power of IBM xSeries 335 can reduce the computational time of high-dimensional data for both methods. Each run had been independently experimented using only one CPU in order to make sure the computational time of every run utilize the same capacity of CPUs usage. This situation was crucial for the performance comparison of computational times between EPSO and BPSO.

**Table 1:** The Description of gene expression data sets

**Data sets**	**Number of samples**	**Number of genes**	**Number of classes**
11_Tumors	174	12,533	11
9_Tumors	60	5,726	9
Brain_Tumor1	90	5,920	5
Brain_Tumor2	50	10,367	4
Leukemia1	72	5,327	3
Leukemia2	72	11,225	3
Lung_Cancer	203	12,600	5
SRBCT	83	2,308	4
Prostate_Tumor	102	10,509	2
DLBCL	77	5,469	2

**Note:** SRBCT = small round blue cell tumor.

DLBCL = diffuse large B-cell lymphomas.

Experimental results that were generated by EPSO were evaluated with an experimental method (BPSO) and other previous PSO-based methods for objective comparisons [3,6-9]. Firstly, a gain ratio technique was applied for pre-processing in order to pre-select 500-top-ranked genes. This technique is a filter-based feature ranking approach and based on the concept of entropy. The original work of the gain ratio technique can be found in Quinlann [17]. The genes were then applied in EPSO and BPSO. Next, the LOOCV classification accuracy on gene subsets that were produced by EPSO and BPSO, was measured by using SVM. Generally, in LOOCV classification accuracy, *N*-1 samples is trained and tested on the remaining sample which has not been used during the training. *N* represents the total number of samples. By cycling through all the samples, we can get realistic and honest estimates of the both approaches. The implementation of LOOCV was in exactly the same way as done by Chuang *et al*. in order to avoid selection bias [6,8,9] where the only one cross-validation cycle (outer loop); namely LOOCV, was utilized, instead of two nested ones. Several experiments were independently carried out 10 times on each data set using EPSO and BPSO. Next, an average result of the 10 independent runs was achieved. To evaluate the performances of EPSO and BPSO, three criteria, following their importance, were considered: LOOCV classification accuracy, the number of selected genes, and computational times. Each criterion should have the best, average, and standard deviation results. High accuracy, the small number of selected genes, and low computational time were needed to obtain an effective performance.

The parameter values for EPSO and BPSO are shown in Table 2. The results of preliminary runs decided the parameter values. The numbers of particles and iterations to reach a good solution are problem dependent [16]. If the numbers were large, both methods would need more time to complete their processes. If the numbers were small, they would take short period of time, but they would not be able to find a good solution. Therefore, we chose intermediate values for the number of particles and iterations between 100 and 500. The value of *w*_1_ was larger than *w*_2_ because the classification accuracy was more important than the number of selected genes. We tried to get the best value based on trial and error approaches. EPSO and BPSO were analyzed using different parameter values. So far, the best values for both *w*_1_ and *w*_2_ using these methods were 0.8 and 0.2, respectively. When *w*_2_ was more than *w*_1_, the number of selected genes also increased. *c*_1_ and *c*_2_ had the same value (2) so that particles would be attracted towards the averages of *Pbest*_*i*_(*t*) and *Gbest*(*t*) [15].

**Table 2:** Parameter settings for EPSO and BPSO

**Parameters**	**Values**
The number of particles	100
The number of iteration (generation)	500
*w*_1_	0.8
*w*_2_	0.2
*c*_1_	2
*c*_2_	2
Cost, *C*	1
Gamma, *g*	1/*k*

Note: *k* = the number of genes (features) in a subset during a training phase in SVM.

In the fitness functions of EPSO and BPSO, SVM classifiers have been used to evaluate the subsets of selected genes. A radial basis function (RBF) kernel was implemented on the SVM classifiers. The rationale to use this kernel was that early results from previous works were readily found with excellent generalization performance in non-linear separable and low computational cost [18]. This RBF kernel nonlinearly maps samples into a higher dimensional space so it, unlike the linear kernel, can handle the case when the relation between class labels and attributes is nonlinear. For a multi-class SVM, we have applied the “one-against-one” approach. Hsu and Lin [19] have provided a detailed comparison and conclude that “one-against-one” is a competitive approach.

### Experimental results

The results yielded by EPSO were almost consistent in all data sets based on the standard deviations in Tables 3 and 4. Attractively, 100% LOOCV accuracy with less than 12 selected genes on the Leukemia1, Leukemia2, and DLBCL data sets had been achieved from all runs. Furthermore, EPSO had efficiently selected and yielded a near-optimal gene subset from high-dimensional data (gene expression data), with a proof that over 92% average classification accuracies had been obtained on other data sets, except for the 9_Tumors data set. Moreover, the standard deviations of the number of selected genes were less than 10 for all of the data sets except for the SRBCT data set (13.03 standard deviations). All the best results achieved 100% LOOCV accuracy with not more than 7 selected genes, indicating that EPSO efficiently selected and produced a near-optimal gene subset from high-dimensional data (gene expression data).

**Table 3:** Experimental result for each using run epso on 11_tumors, 9_tumors, brain_tumor1, brain_tumor2 and leukemia1 data sets

**Run#**	**11_Tumors**		**9_Tumors**		**Brain_Tumor1**		**Brain_Tumor2**		**Leukemia1**	
	**#Acc (%)**	**#Selected genes**	**#Acc (%)**	**#Selected genes**	**#Acc (%)**	**#Selected genes**	**#Acc (%)**	**#Selected genes**	**#Acc (%)**	**#Selected genes**
1	**96.55**	**243**	**76.67**	**251**	**93.33**	**8**	**94**	**4**	100	3
2	95.98	245	76.67	255	93.33	11	94	5	100	4
3	95.98	250	75	231	92.22	6	94	8	100	3
4	95.40	232	75	237	92.22	7	92	4	100	3
5	95.40	241	75	242	92.22	8	92	7	100	3
6	95.40	244	75	253	92.22	9	92	7	100	4
7	94.83	218	75	255	92.22	11	92	7	100	4
8	94.83	229	75	261	91.11	3	92	7	100	3
9	94.83	232	73.33	238	91.11	5	92	8	100	3
10	94.83	243	73.33	248	91.11	7	90	3	**100**	**2**
Average ± S.D.	95.40 ±0.61	237.70 ±9.66	75 ±1.11	247.10 ±9.65	92.11 ±0.82	7.5 ±2.51	92.40 ±1.26	6.00 ±1.83	100.00 ±0	3.20 ±0.63

**Note**: Results of the best subsets are written in a **bold** style. A near-optimal subset that produces the highest classification accuracy with the smallest number of genes is selected as the best subset. #Acc and S.D. denote the classification accuracy and the standard deviation, respectively, whereas #Selected Genes and Run# represent the number of selected genes and a run number, respectively.

**Table 4:** Experimental results for each run using epso on leukemia2, lung_cancer, SRBCT prostate_tumor, and DLBCL data stes

**Run#**	**Leukemia2**		**Lung_Cancer**		**SRBCT**		**Prostate_Tumor**		**DLBCL**	
	**#Acc (%)**	**#Selected genes**	**#Acc (%)**	**#Selected genes**	**#Acc (%)**	**#Selected genes**	**#Acc (%)**	**#Selected genes**	**#Acc (%)**	**#Selected genes**
1	**100**	**4**	**96.06**	**7**	100	27	**99.02**	**5**	**100**	**3**
2	100	4	96.06	10	100	11	98.04	4	100	4
3	100	5	96.06	12	100	12	98.04	6	100	4
4	100	6	95.57	6	98.80	8	98.04	8	100	5
5	100	7	95.57	7	98.80	9	98.04	8	100	5
6	100	7	95.57	7	100	48	98.04	11	100	5
7	100	7	95.57	8	98.80	7	98.04	8	100	5
8	100	8	95.57	9	100	12	97.06	4	100	5
9	100	9	95.57	11	100	8	97.06	6	100	5
10	100	11	95.07	6	**100**	**7**	97.06	6	100	6
Average ± S.D.	100.00 ±0	6.80 ±2.20	*95.67 ±0.31*	*8.30 ±2.11*	99.64 ±0.58	14.90 ±13.03	97.84 ±0.62	6.60 ±2.17	100.00 ±0	4.70 ±0.82

**Note**: Results of the best subsets shown are written in a **bold** style. A near-optimal subset that produces the highest classification accuracy with the smallest number of genes is selected as the best subset. #Acc and S.D. denote the classification accuracy and the standard deviation, respectively, whereas #Selected Genes and Run# represent the number of selected genes and a run number, respectively.

Practically, the best subset of a data set is first chosen and the genes in it are then listed for biological usage. These informative genes, among thousands of genes, may be excellent candidates for clinical and medical investigations. Biologists can save time because they can directly refer to the genes that have higher possibilities of being useful for cancer diagnoses and as drug targets in the future. The best subset is chosen based on the highest classification accuracy with the smallest number of selected genes. The highest accuracy provides confidence for the most accurate classification of cancer types. Moreover, the smallest number of selected genes for cancer classification can reduce the cost in clinical settings.

**Figure 2:**
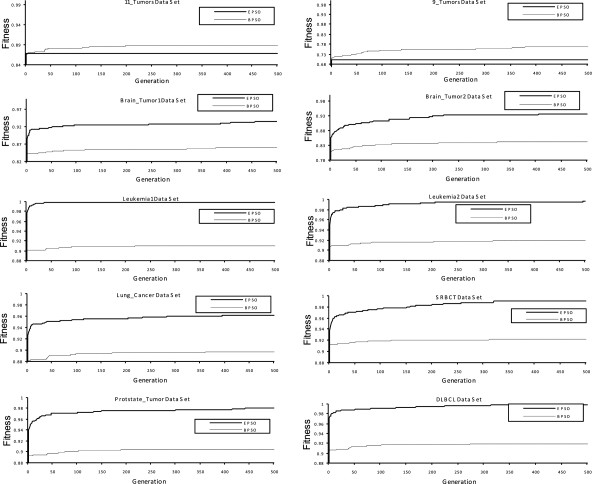
The relation between the average of fitness values (10 runs on average) and the number of generations for EPSO and BPSO.

The averages of fitness values of EPSO increased dramatically after a few generations on all the data sets as shown in Figure 2. This trend proved that EPSO is suitable for selecting a small number of genes from high-dimensional data (gene expression data) to maximize classification accuracy. It also proved that a combination between a high classification rate and a small number (subset) of selected genes can produce a high fitness value. The condition of the proposed particles’ speed that should always be positive real numbers started in the initialization method, the modified sigmoid function, and the new rule for updating particle’s positions, which provoked the early convergence of EPSO. The fitness value of EPSO still increased for further generations on all the data sets except for 9_Tumors and 11_Tumors data sets. However, there was only a small rise in values. EPSO did the exploration in order to find a good solution and it had been proven by this growth. In contrast, *P*(*x*_*i*_^*d*^(*t*) = 0) = *P*(*x*_*i*_^*d*^(*t*) = 1) = 0.5. caused the averages of fitness values of BPSO to become lower than the fitness value of EPSO until the last generation, except for the 9_Tumors and 11_Tumors data sets.

In general, it is worthwhile to mention that the classification accuracy, the number of selected genes, and computational times of EPSO were superior to BPSO in terms of the best, average, and standard deviation results on all the data sets except for the 9_Tumors data set according to the result in Table 5. The computational times of BPSO were also higher than EPSO in all the data sets. EPSO was able to minimize its computational times because of the following reasons:

1) EPSO chose smaller number of genes compared to BPSO;

2) SVM utilized a small number of features (genes) that were selected by EPSO for the classification process which caused the computation of SVM to be fast;

3) Only the speed of a particle for comparing with *r*_3_^*d*^(*t*) was used by EPSO, whereas all elements of a particle’s velocity vector for the comparison had been practiced by BPSO.

**Table 5:** Comparative Experimental Results of the best subsets produced by EPSO and BPSO

**Data**	**Method**	**EPSO**			**BPSO**		
	**Evaluation**	**Best**	**#Ave**	**S.D**	**Best**	**#Ave**	**S.D**
11_Tumors	#Acc (%)	96.55	95.40	**0.61**	**97.7**	**96.78**	0.73
	#Genes	243	237.70	**9.66**	**230**	**221.30**	14.48
	#Time	**67.21**	**67.05**	**0.22**	212.44	252.05	32.87
9_Tumors	#Acc (%)	76.67	75.00	**1.11**	**85**	**82.67**	2.25
	#Genes	251	247.10	9.65	**250**	**244.30**	**9.43**
	#Time	**4.35**	**4.29**	**0.09**	17.97	17.74	0.10
Brain_Tumor1	#Acc (%)	**93.33**	92.11	0.82	**93.33**	**92.44**	**0.47**
	#Genes	**8**	**7.5**	**2.51**	217	215	7.24
	#Time	**14.89**	**14.82**	**0.12**	24.62	24.76	0.15
Brain_Tumor2	#Acc (%)	**94**	**92.4**	1.27	92	91.40	**0.97**
	#Genes	**4**	**6.0**	**1.83**	208	225.20	13.13
	#Time	**0.85**	**0.85**	**0.002**	6	6.08	0.09
Leukemia1	#Acc (%)	**100**	**100**	**0**	**100**	98.75	0.44
	#Genes	**2**	**3.20**	**0.63**	213.00	198.20	6.48
	#Time	**2.80**	**2.79**	**0.01**	6.97	6.99	0.05
Leukemia2	#Acc (%)	**100**	**100**	**0**	**100**	**100**	**0**
	#Genes	**4**	**6.8**	**2.2**	195	200.80	4.24
	#Time	**3.1**	**3.1**	**0.02**	10.05	10.16	0.15
Lung_Cancer	#Acc (%)	96.06	95.67	0.31	**98.03**	**97.73**	**0.25**
	#Genes	**7**	**8.3**	**2.11**	212	212.90	9.26
	#Time	**123.95**	**124.08**	**0.49**	145.98	145.93	1.00
SRBCT	#Acc (%)	**100**	99.64	0.58	**100**	**100**	**0**
	#Genes	**7**	**14.90**	13.03	191	195.30	**3.23**
	#Time	**7.51**	**7.51**	**0.03**	22.75	22.63	0.22
Prostate_Tumor	#Acc (%)	**99.02**	97.84	0.62	98.04	**98.04**	**0.00**
	#Genes	**5**	**6.6**	**2.17**	195	199.40	3.41
	#Time	**4.37**	**4.37**	**0.008**	22.57	22.27	0.16
DLBCL	#Acc (%)	**100**	**100**	**0**	**100**	**100**	**0**
	#Genes	**3**	**4.7**	**0.82**	193	200.90	3.78
	#Time	**1.99**	**1.99**	**0.008**	7.72	7.75	0.10

**Note**: The best result of each evaluation is written in a **bold** style. The best method of each data set is shown in the shaded cells. The best method is selected based on the highest number of the best results for all evaluations. #Acc and S.D. denote the classification accuracy and the standard deviation, respectively, whereas #Genes and #Ave represent the number of selected genes and an average, respectively. #Time stands for running time in the hour unit.

We compared our work with previous related works that used PSO-based methods in their proposed methods for an objective comparison [3,6-9]. Table 6 shows the result of the comparison. Two criteria were used to evaluate the performance of EPSO and the other methods: classification accuracy and the number of selected genes. Higher accuracy with a smaller number of selected genes is needed to obtain superior performance. For all of the data sets, the averages of the number of the selected genes for our work were smaller than the previous works [3,6-9]. Our work also produced higher classification accuracies on the six data sets (Brain_Tumor2, Leukemia1, Leukemia2, SRBCT, Prostate_Tumor, and DLBCL) compared to the previous PSO-based methods [3,6-9]. Moreover, our method resulted in higher averages of the classification accuracies on all data sets compared to the other methods [3,7]. The number of selected genes of our work in terms of average and the best results were also smaller than the previous works for all the data sets. However, Chuang *et al*. showed better classification accuracies than our work on four data sets (11_Tumors, 9_Tumors, Brain_Tumor1, and Lung_Cancer) [8].

**Table 6:** A comparison between our method (EPSO) and previous PSO-based methods

**Data**	**Method**	**EPSO [This work]**	**IBPSO**[6]	**PSOTS**[3]	**PSOGA**[7]	**TS-BPSO**[8]	**BPSO-CGA**[9]
	**Evaluation**						
11_Tumors	Average #Acc (%)	95.40	-	-	-	-	-
	Best #Acc (%)	96.55	93.10	-	-	**97.35**	-
	Average #Genes	237.70	-	-	-	-	-
	Best #Genes	**243**	2948	-	-	3206	-
9_Tumors	Average #Acc (%)	75	-	-	-	-	-
	Best #Acc (%)	76.67	78.33	-	-	**81.63**	-
	Average #Genes	247.10	-	-	-	-	-
	Best #Genes	**251**	1280	-	-	2941	-
Brain_Tumor1	Average #Acc (%)	92.11	-	-	-	-	
	Best #Acc (%)	93.33	94.44	-	-	**95.89**	91.4
	Average #Genes	7.5	-	-	-	-	-
	Best #Genes	**8**	754	-	-	2913	456
Brain_Tumor2	Average #Acc (%)	92.4	-	-	-	-	-
	Best #Acc (%)	**94**	**94.00**	-	-	92.65	-
	Average #Genes	6.0	-	-	-	-	-
	Best #Genes	**4**	1197	-	-	5086	-
Leukemia1	Average #Acc (%)	**100**	-	98.61	95.10	-	-
	Best #Acc (%)	**100**	**100**	-	-	**100**	**100**
	Average #Genes	**3.2**	-	7	21	-	-
	Best #Genes	**2**	1034	-	-	2577	300
Leukemia2	Average #Acc (%)	100	-	-	-	-	-
	Best #Acc (%)	**100**	**100**	-	-	**100**	-
	Average #Genes	6.8	-	-	-	-	-
	Best #Genes	**4**	1292	-	-	5609	-
Lung_Cancer	Average #Acc (%)	95.67	-	-	-	-	-
	Best #Acc (%)	96.06	96.55	-	-	**99.52**	-
	Average #Genes	8.3	-	-	-	-	-
	Best #Genes	**7**	1897	-	-	6958	-
SRBCT	Average #Acc (%)	99.64	-	-	-	-	-
	Best #Acc (%)	**100**	**100**	-	-	**100.00**	**100**
	Average #Genes	14.90	-	-	-	-	-
	Best #Genes	**7**	431	-	-	1084	880
Prostate_Tumor	Average #Acc (%)	97.84	-	-	-	-	-
	Best #Acc (%)	**99.02**	92.16	-	-	95.45	93.7
	Average #Genes	6.6	-	-	-	-	-
	Best #Genes	**5**	1294	-	-	5320	795
DLBCL	Average #Acc (%)	100	-	-	-	-	-
	Best #Acc (%)	**100**	**100**	-	-	**100.00**	-
	Average #Genes	4.7	-	-	-	-	-
	Best #Genes	**3**	1042	-	-	2671	-

**Note**: The best result of each evaluation is written in a **bold** style. The best result of evaluations could not be compared and stated if the results of evaluations have not been reported in the previous works. The best method of each data set is shown in the shaded cells. The best method is selected based on the highest number of the best results for all evaluations. #Acc and S.D. denote the classification accuracy and the standard deviation, respectively, whereas #Genes and #Ave represent the number of selected genes and an average, respectively. #Time stands for running time in the hour unit. ‘-‘ means that a result is not reported in the previous related work.

IBPSO = An improved binary PSO. PSOTS = A hybrid of PSO and tabu search.

PSOGA = A hybrid of PSO and GA. TS-BPSO = A combination of tabu search and BPSO.

BPSO-CGA = A hybrid of BPSO and a combat genetic algorithm.

The most recent works came up with similar LOOCV results (100%) with ours on the Leukemia1, Leukemia2, SRBCT, and DLBCL data sets, but they used more than 400 genes to obtain these results [6,8,9]. Moreover, their experimental results were obtained by utilizing only one independent run on each data set and not based on average results, causing that they could not have statistically meaningful conclusions. Since the proposed method is a stochastic approach, therefore the average results are important. Overall, our work outperformed the other methods in terms of the LOOCV accuracy and the number of selected genes on six data sets (Brain_Tumor2, Leukemia1, Leukemia2, SRBCT, Prostate_Tumor, and DLBCL). The running times between EPSO and these works could not be compared because they were not reported.

According to Figure 2 and Tables 3–6, EPSO is reliable for gene selection where it had yielded the near-optimal solution from gene expression data. This was due to the proposed particles’ speed, the modified sigmoid function, and the introduced rule which maximized the probability *x*_*i*_^*d*^(*t* + 1) = 0 (*P*(*x*_*i*_^*d*^(*t* + 1) = 0)). This high probability caused the selection of a small number of informative genes and finally produced a near-optimal subset (a small subset of informative genes with high classification accuracy) for cancer classification. The particles’ speed was introduced to provide the rate at which a particle changed its position, whereas the rule was proposed to update the particle’s positions. The sigmoid function was modified for increasing the probability of bits in particle’s positions to be zero.

## Conclusions

In this paper, EPSO is proposed for gene selection on ten gene expression data sets. In general, the performance of EPSO is better than BPSO and PSO-based methods that have been proposed in previous related works, in terms of classification accuracy and the number of selected genes. EPSO is efficient because the probability *x*_*i*_^*d*^(*t* + 1) = 0 is increased by the particle speed, the modified sigmoid function, and the introduced rule in order to yield a near-optimal subset of genes for better cancer classification. EPSO also features lower running times because it selects only a smaller number of genes compared to BPSO. A modified representation of particle’s positions in PSO will be proposed to minimize the number of genes subsets in solution spaces for future works

## Competing interests

The authors declared that they have no competing interests.

## Authors’ contributions

MSM is a principal researcher and proposed the main idea for this study. SO, SD, MY supported and checked the manuscript. Moreover, they gave well advises. AA and ZI aided in interpretation of the results. All authors read and approved the final manuscript.

## References

[B1] Knudsen S A biologist’s guide to analysis of DNA microarray data 2002 New York, USA: John Wiley & Sons

[B2] Mohammad MS Omatu S Yoshioka M Deris S **A cyclic hybrid method to select a smaller subset of informative genes for cancer classification**, *International Journal of Innovative Computing* Inf Control 2009 8 8 2189 2202

[B3] Shen Q Shi WM Kong W Hybrid particle swarm optimization and tabu search approach for selecting genes for tumor classification using gene expression data Comput Biol Chem 2009 8 53 60 10.1016/j.compbiolchem.2007.10.001 18093877

[B4] Pavlidis SP Payne AM Swift SM Multi-membership gene regulation in pathway based microarray analysis Algorithms for Molecular Biology 2011 8 22 10.1186/1748-7188-6-22 21939531 PMC3189100

[B5] Liu Z Magder LS Hyslop T Mao L Survival associated pathway identification with group L_P_ penalized global AUC maximization Algorithms for Molecular Biology 2010 8 30 10.1186/1748-7188-5-30 20712896 PMC2930641

[B6] Chuang LY Chang HW Tu CJ Yang CH Improved binary PSO for feature selection using gene expression data Comput Biol Chem 2009 8 29 38 10.1016/j.compbiolchem.2007.09.005 18023261

[B7] Li S Wu X Tan M Gene selection using hybrid particle swarm optimization and genetic algorithm Soft Computing 2008 8 1039 1048 10.1007/s00500-007-0272-x

[B8] Chuang LY Yang CH Yang CH Tabu search and binary particle swarm optimization for feature selection using microarray data J Comput Biol 2009 8 12 1689 1703 10.1089/cmb.2007.0211 20047491

[B9] Chuang LY Yang CH Li JC Yang CH A hybrid BPSO-CGA approach for gene selection and classification of microarray data J Comput Biol 2011 8 1 14 10.1089/cmb.2010.0102 21210743 PMC3244808

[B10] Kennedy J Eberhart R Particle swarm optimization 1995 Perth, Australia: Proc. 1995 IEEE Int. Conf. Neural Networks 4, IEEE Press 1942 1948

[B11] Yao B Li S ANMM4CBR A case-based reasoning method for gene expression data classification Algorithms For Molecular Biology 2010 8 14 10.1186/1748-7188-5-14 20051140 PMC2843690

[B12] Kennedy J Eberhart R A discrete binary version of the particle swarm algorithm Proc. 1997 IEEE Int. Conf. Systems, Man, and Cybernetics, IEEE Press, Florida, USA 1997 8 4104 4108

[B13] Shi Y Eberhart RC A modified particles swarm optimizer 1998 Piscataway, NJ: Proc. 1998 IEEE Congress on Evolutionary Computation, IEEE Pres 69 73

[B14] Naka S Genji T Yura T Fukuyama Y Practical distribution state estimation using hybrid particle swarm optimization 2001 Ohio, USA: Proc. 2001 IEEE Power Engineering Society Winter Meeting, IEEE Press 815 820

[B15] Peram T Veeramacheneni K Mohan CK Fitness-distance-ratio based particle swarm optimization 2003 Indiana, USA: Proc. 2003 IEEE Swarm Intelligence Symposium, IEEE Press 174 181

[B16] Engelbrecht AP Fundamentals of computational swarm intelligence 2005 West Succex, England: John Wiley and Sons

[B17] Quinlan JR Induction of decision tress Mach Learn 1986 8 1 81 106

[B18] Suykens JAK Vandewalle J Least squares support vector machine classifiers Neural Processing Letters 1999 8 3 293 300 10.1023/A:1018628609742

[B19] Hsu CW Lin CJ A comparison of methods for multi-class support vector machines IEEE Transaction on Neural Networks 2002 2002 8 2 415 425 10.1109/72.991427 18244442

